# Bone remodeling: a central mechanism in prostate cancer bone metastasis

**DOI:** 10.7717/peerj.21344

**Published:** 2026-06-09

**Authors:** Hao Tang, Jun Chen, Honghan Wu, Yuanhao Lv, Qingde Wa, Sisi He

**Affiliations:** 1 Department of Orthopedic Surgery, The Second Affiliated Hospital of Zunyi Medical University, Zunyi, Guizhou, China; 2 Department of Oncology, The Second Affiliated Hospital of Zunyi Medical University, Zunyi, Guizhou, China

**Keywords:** Prostate cancer bone metastasis, Bone remodeling, Osteoblasts, Osteoclasts, Osteocytes, Tumor-bone microenvironment interactions, Mechanism

## Abstract

Prostate cancer (PCa) commonly metastasizes to bone, leading predominantly to osteoblastic lesions driven by intricate cellular interactions within the bone microenvironment. While osteoclasts (OCLs) initiate bone remodeling through resorption, their contribution to PCa progression appears limited, as pharmacological inhibition with bisphosphonates and RANKL antagonists yields only modest clinical benefit. In contrast, osteoblasts (OBs) exert dual roles, either promoting or restraining tumor growth through context-dependent signaling pathways, including Wnt5a-mediated dormancy and transforming growth factor-beta (TGF-β)–induced epithelial–mesenchymal transition (EMT). Bone-derived growth factors such as insulin-like growth factor I/II (IGF-I/II), fibroblast growth factor 23 (FGF-23), and platelet-derived growth factor (PDGF) further enhance tumor colonization. Osteocytes (OCYs), the most abundant and long-lived bone cells, directly interact with PCa cells and, in response to altered mechanotransduction, release pro-metastatic mediators including CCL5 and matrix metalloproteinases (MMPs). Moreover, PCa cells actively reprogram the bone niche by secreting exosomes and paracrine factors such as parathyroid hormone–related peptide (PTHrP) and Wnt7b, driving OBs and OCYs toward tumor-supportive phenotypes. Together, these reciprocal interactions establish a self-reinforcing cycle of bone remodeling and tumor progression. This review underscores the central role of bone remodeling in PCa bone metastasis and highlights promising therapeutic targets within the PCa–bone axis.

## Introduction

Prostate cancer (PCa) is the second most common malignancy among men worldwide, accounting for approximately 13.5% of male cancer cases, and its incidence continues to rise alongside socioeconomic development (Taveira-Barbosa et al., 2025; Zhao et al., 2025). Although surgery and radiotherapy achieve favorable outcomes in localized disease, many patients ultimately develop recurrence and progress to castration-resistant prostate cancer (CRPC), which frequently metastasizes to bone. Skeletal metastases are a major cause of morbidity and mortality and substantially compromise both survival and quality of life. Current therapies for advanced disease remain limited in efficacy, underscoring the need for improved mechanistic insights and more effective therapeutic strategies (Baldessari et al., 2023; Harsini et al., 2023; Le et al., 2023; Ayzman, Pachynski & Reimers, 2025).

Bone remodeling under physiological conditions is a dynamic and tightly regulated process that balances bone resorption and bone formation (Hu et al., 2025). This process is coordinated within basic multicellular units (BMUs), which traditionally describe coupled osteoclast–osteoblast teams; osteocytes (OCYs) regulate these units by integrating mechanical and biochemical cues to coordinate resorption and formation (Woo et al., 2025). Osteoblasts (OBs), derived from mesenchymal stem cells, synthesize and mineralize bone matrix (Wang et al., 2025b). Osteoclasts (OCLs), originating from hematopoietic stem cells under macrophage-lineage influence, differentiate into multinucleated cells specialized for matrix degradation (McDonald et al., 2021). Together, these cell populations maintain skeletal homeostasis, with approximately 10% of the human skeleton undergoing turnover each year (Singh et al., 2023; Martin & Seeman, 2023).

Osteocytes, the most abundant and long-lived bone cells, form an extensive three-dimensional network through dendritic extensions. They sense mechanical loading and fluid shear stress and translate these stimuli into biochemical signals that regulate osteoblast and osteoclast activity. Key pathways include the OPG/RANKL axis, which modulates osteoclastogenesis, and the SOST/Dkk1/WNT pathway, which influences osteoblast differentiation (Galea, Lanyon & Price, 2017; Hansdah & Lui, 2024; Yuze et al., 2025).

**Figure 1:**
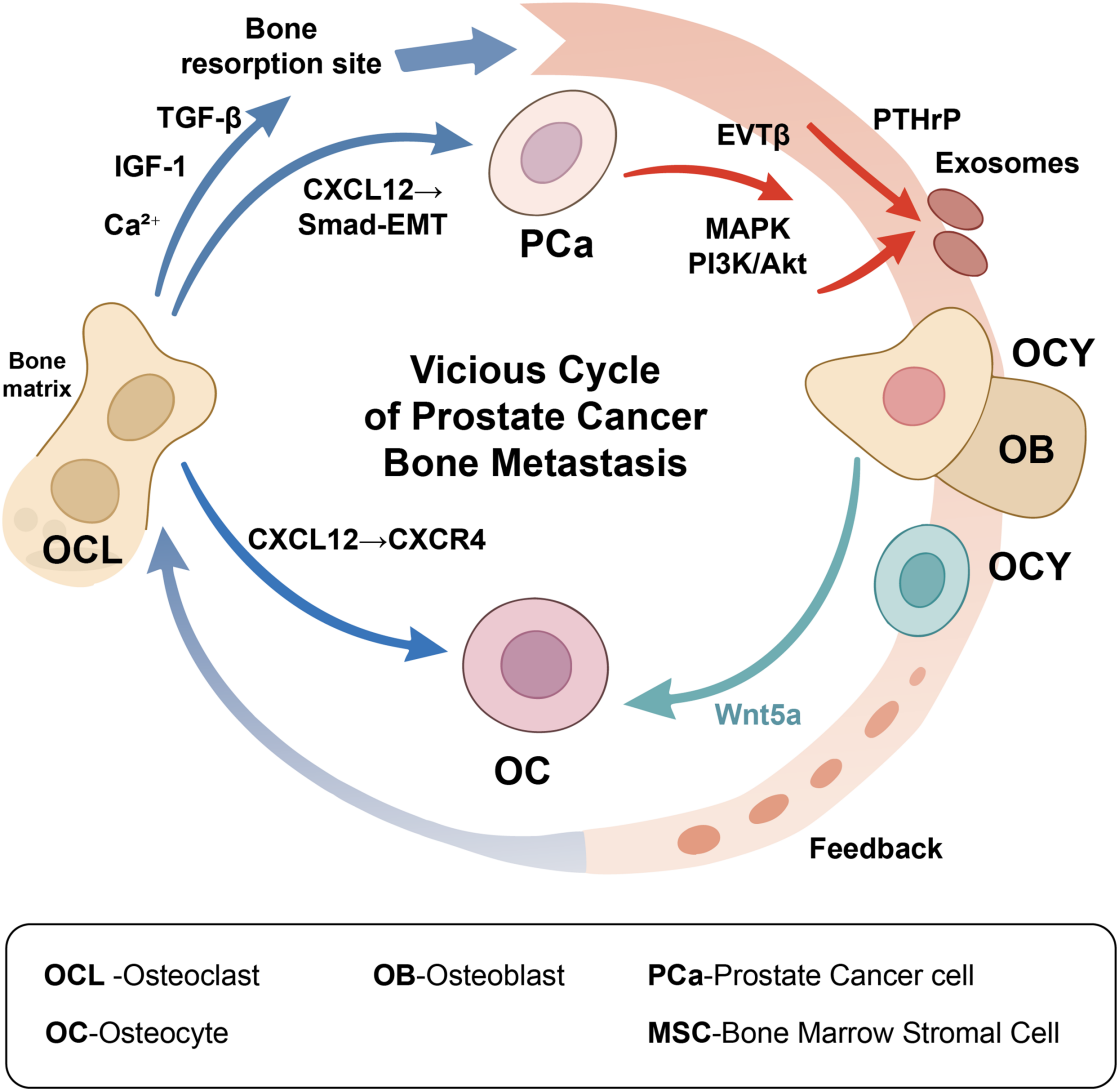
Vicious cycle of bone remodelling in prostate cancer bone metastasis. Prostate cancer (PCa) cells home to sites of active bone remodelling and establish reciprocal interactions with osteoclasts (OCLs), osteoblasts (OBs), and osteocytes (OCYs). Osteoclastic resorption releases bone matrix-derived factors, including transforming growth factor-beta (TGF-β), IGF-1, and Ca^2+^, which promote tumour colonisation and Smad-mediated epithelial–mesenchymal transition. Osteoblast- and osteocyte-associated CXCL12/CXCR4 signalling supports tumour homing and niche retention, whereas tumour-derived factors, including ETV1, parathyroid hormone–related peptide (PTHrP), and exosomes, activate mitogen-activated protein kinase (MAPK) and PI3K/Akt signalling and stimulate osteoblastic responses. Osteocyte-derived Wnt5a contributes to feedback regulation within the metastatic niche, together forming a self-reinforcing cycle of tumour growth in bone.

Figure 1 illustrates the vicious cycle of prostate cancer bone metastasis. When cancer metastasizes to bone, the balance between osteoclastic resorption and osteoblastic formation is disrupted (Shupp et al., 2018). Bone metastases are traditionally classified as osteoblastic or osteolytic. PCa metastases are predominantly osteoblastic and are characterized by aberrant new bone formation that is structurally abnormal and biologically defective (Archer Goode, Wang & Munkley, 2023). Paradoxically, despite this osteoblastic phenotype, elevated bone turnover markers indicate that osteoclastic resorption is also increased (Elaasser, Arakil & Mohammad, 2024). This apparent contradiction underscores the complex coupling between bone formation and resorption in PCa skeletal metastases.

Understanding how osteoblasts, osteoclasts, and osteocytes are reprogrammed within the metastatic niche is central to clarifying the pathophysiology of PCa bone metastasis. Reciprocal interactions between tumor cells and bone-resident cells establish a self-reinforcing cycle in which tumor-derived signals drive abnormal bone remodeling, and bone-derived factors released during remodeling further promote tumor colonization and growth. Elucidating these cellular and molecular mechanisms offers critical opportunities to disrupt the PCa–bone axis and improve outcomes for patients with skeletal metastases.

## Target audience

This review is intended for researchers, clinicians, and graduate students specializing in oncology, orthopedics, and bone biology. It will also be of interest to translational scientists and healthcare professionals focused on prostate cancer progression, skeletal metastasis, and tumor–bone microenvironment interactions. By synthesizing recent mechanistic insights and therapeutic perspectives, this article aims to serve both as a foundational resource for early-career investigators and as an up-to-date reference for experts engaged in prostate cancer research and management.

## Survey Methodology

This review synthesizes literature published between 2000 and 2025, retrieved primarily from PubMed (https://pubmed.ncbi.nlm.nih.gov/) and Web of Science (https://www.webofscience.com/). The search strategy combined Medical Subject Headings (MeSH) with relevant free-text terms. Keywords included “prostate cancer,” “bone metastasis,” “osteoblasts,” “osteoclasts,” “osteocytes,” “bone microenvironment,” “tumor dormancy,” “bone remodeling,” “CXCL12/CXCR4 axis,” “RANKL,” “PTHrP,” and “Wnt signaling.”

Initial screening was conducted through evaluation of article titles and abstracts. Eligible studies comprised mechanistic investigations, *in vivo* and *in vitro* experimental research, and high-quality reviews directly addressing prostate cancer bone metastasis. Full-text assessments were subsequently performed to ensure inclusion of peer-reviewed publications that provided substantive insights into the cellular and molecular mechanisms governing tumor–bone interactions.

## Physiological bone remodeling

Bone remodeling is a continuous process that preserves skeletal integrity through two tightly coupled phases: bone resorption, mediated by osteoclasts, and bone formation, carried out by osteoblasts (Moura et al., 2025; Daponte, Henke & Drissi, 2024). These processes occur sequentially within a remodeling cycle comprising five phases: activation, resorption, reversal, formation, and termination (Adejuyigbe et al., 2023).

Three principal cell types orchestrate this cycle. Osteoclasts degrade existing bone matrix to initiate resorption, osteoblasts synthesize and mineralize new matrix, and osteocytes—embedded within the mineralized bone matrix—regulate remodeling by sensing mechanical cues and maintaining mineral homeostasis (Marahleh et al., 2023; Choi et al., 2024).

The remodeling process is further modulated by systemic and local factors, including parathyroid hormone (PTH), calcitriol (the active form of vitamin D), bone morphogenetic proteins (BMPs), transforming growth factor-β (TGF-β), growth hormone, glucocorticoids, thyroid hormones, and sex steroids (Chen et al., 2024). Through diverse signaling pathways, these regulators fine-tune osteoblast and osteoclast activity to preserve skeletal balance.

**Figure 2:**
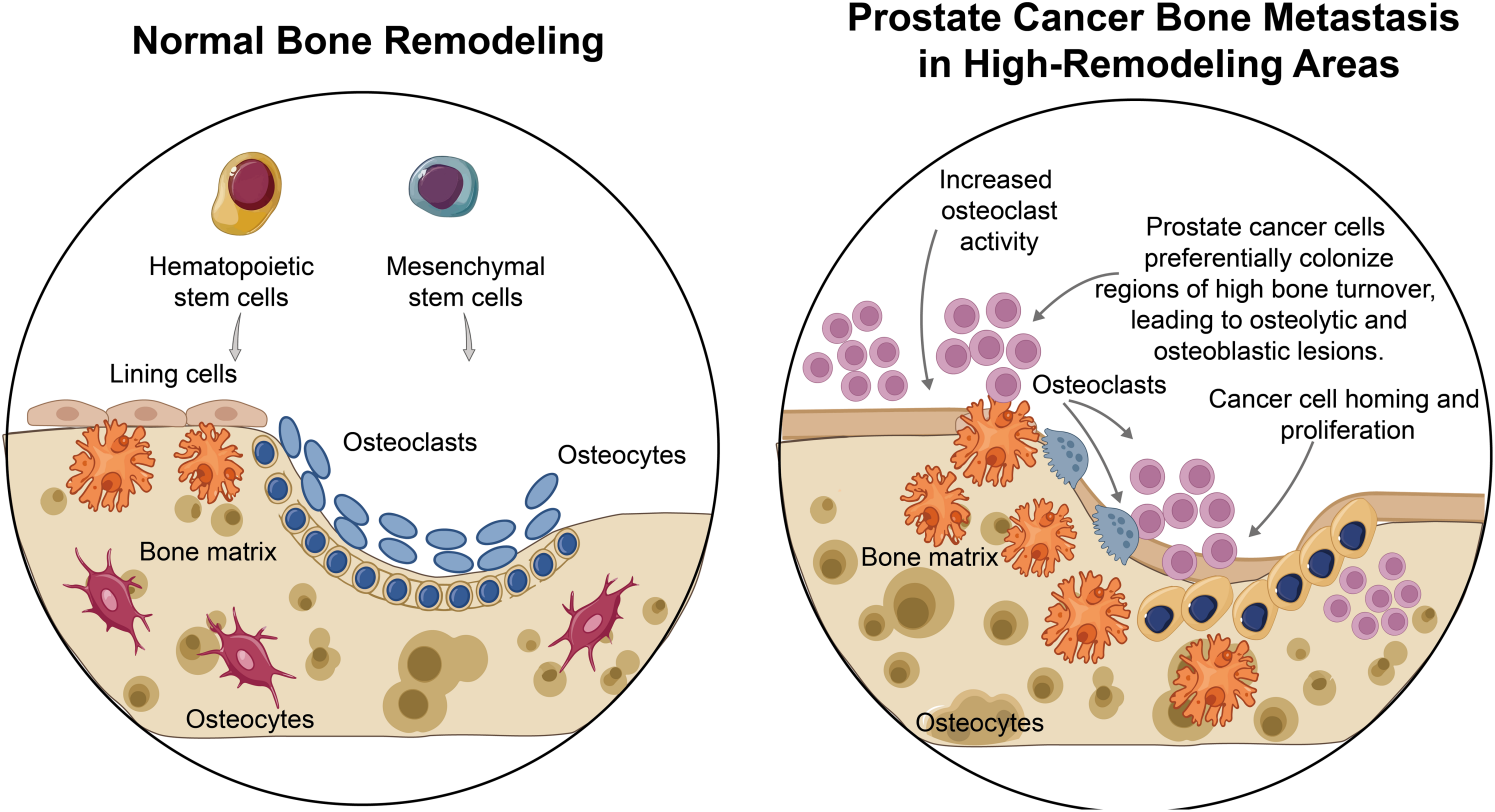
Bone structure and remodeling: roles of stem cells and bone cells. Normal bone remodeling *vs* prostate cancer bone metastasis in high-remodeling regions. The left panel illustrates physiological bone remodeling, in which hematopoietic stem cells (HSCs) and mesenchymal stem cells (MSCs) provide precursors for osteoclasts and osteoblast-lineage cells. Osteoclast-mediated bone resorption occurs at the bone surface beneath lining cells, while osteocytes embedded in the bone matrix coordinate remodeling to maintain skeletal homeostasis. The right panel depicts the bone microenvironment during prostate cancer bone metastasis in areas of high turnover. Prostate cancer cells preferentially home to and colonize these regions, where enhanced osteoclast activity and dysregulated osteoblast responses disrupt the remodeling balance, promoting tumor cell proliferation and resulting in mixed osteolytic and osteoblastic lesions.

In the context of prostate cancer bone metastasis, this physiological equilibrium is profoundly disrupted. The normal coupling between resorption and formation becomes dysregulated, leading to pathological bone formation accompanied by compromised structural integrity within the metastatic niche. Figure 2 contrasts normal bone remodeling with prostate cancer bone metastasis in high turnover areas.

## Prostate cancer preferentially metastasizes to skeletal sites with high remodeling activity

Under physiological conditions, bone remodeling depends on a finely tuned balance between osteoclastic resorption and osteoblastic formation (Moura et al., 2025). Metastatic infiltration disrupts this equilibrium: PCa-derived signals and their crosstalk with bone-resident cells dysregulate remodeling dynamics, driving a self-sustaining cycle of pathological bone formation and tumor expansion (Kim et al., 2025).

Skeletal metastases occur most frequently in regions with high turnover, including the vertebrae, ribs, pelvis, and proximal long bones (Cao et al., 2025). These high-remodeling areas provide fertile “soil” for metastatic seeding (Archer Goode, Wang & Munkley, 2023). Experimental studies support this clinical pattern: young mice with elevated remodeling activity (7 weeks old) develop greater skeletal tumor burdens after PCa inoculation than older mice (52 weeks old). Similarly, pharmacological enhancement of bone turnover facilitates tumor colonization. Notably, in murine models, PCa cells preferentially home to the lateral tibia, which exhibits significantly higher remodeling rates than the medial side (Elaasser, Arakil & Mohammad, 2024). Within these microdomains, reciprocal interactions among tumor cells, osteoblasts, and osteoclasts establish a feed-forward loop that accelerates both tumor progression and metastatic outgrowth (Wang et al., 2019). Below, we summarize key determinants of skeletal tropism, including marrow vascular accessibility, chemokine- and adhesion-mediated homing, and remodeling-derived trophic cues.

### Mechanistic basis for skeletal tropism and homing in prostate cancer

PCa metastasis to bone follows a multistep cascade that includes tumor cell transformation, angiogenesis, invasive growth, dissemination from the primary tumor, and colonization of distant skeletal sites (Archer Goode, Wang & Munkley, 2023). During this process, circulating tumor cells interact with vascular endothelial cells, extravasate through the vessel wall, and establish site-specific lesions (Tang et al., 2025). Although bone is a mineralized tissue that imposes physical and metabolic barriers to colonization (Elaasser, Arakil & Mohammad, 2024), PCa cells adapt to and reprogram the microenvironment to support survival. By engaging bone-resident cells, they secrete factors that stimulate osteoclastic resorption, thereby creating niches favorable for adhesion, proliferation, and metastatic expansion (Jiang, 2025). Bone marrow sinusoids, particularly in metaphyseal regions, exhibit relatively discontinuous endothelial features that can facilitate tumor cell arrest and extravasation from the circulation (Chen, Han & Kang, 2021). Prostate cancer exhibits a marked propensity to colonize the skeleton, a preference that is often most evident in regions with active remodeling and abundant marrow spaces (Wong et al., 2019; Archer Goode, Wang & Munkley, 2023). Mechanistically, this tropism is best explained by convergence of: (i) permissive vascular access and supportive niche architecture within the bone marrow (Kusumbe, 2016), (ii) chemokine- and adhesion-mediated homing cues that promote tumor cell arrest and retention (Ullah, 2019), and (iii) remodeling-driven release of trophic factors that support survival and early outgrowth (Buijs, Stayrook & Guise, 2011; Trivedi et al., 2021). In particular, metaphyseal and trabecular-rich compartments provide extensive marrow sinusoids with relatively discontinuous endothelial features, which can facilitate extravasation and early lodging compared with less vascularized cortical compartments (Batoon & McCauley, 2021). Thus, “high turnover” is not a standalone attractor; rather, turnover is coupled to marrow vascularity and niche availability, together creating a microenvironment that is both accessible and supportive for disseminated tumor cells.

A central molecular axis frequently implicated in PCa skeletal homing is the CXCL12/CXCR4 pathway, which can guide circulating tumor cells toward CXCL12 produced by bone marrow stromal and osteoblast-lineage cells and promote retention within endosteal and perivascular niches (Sharma et al., 2022). Adhesion interactions (*e.g*., selectins and integrins, together with endothelial adhesion molecules) further stabilize tumor cell arrest and transendothelial migration. Once seeded, remodeling-associated signals can amplify tumor fitness: osteoclast-mediated resorption releases matrix-stored growth factors (*e.g*., TGF-β and insulin-like growth factor (IGFs)), while osteoblast-lineage cells provide additional paracrine support. Collectively, these processes enable a “seed-and-soil” loop in which bone turnover and tumor-driven remodeling reinforce one another (Archer Goode, Wang & Munkley, 2023). This framework also helps reconcile clinical observations that predominantly osteoblastic lesions may still exhibit elevated turnover markers, reflecting concurrent—yet pathologically uncoupled—resorption and formation within the metastatic niche (Archer Goode, Wang & Munkley, 2023).

Spatially, emerging experimental evidence suggests that early PCa lodging can vary between cortical and trabecular/endocortical regions depending on local niche composition, osteoblast-lineage cell distribution, and microvascular organization (Wang et al., 2014; Wells et al., 2023). Endosteal surfaces may provide direct access to osteoblast-lineage cues that regulate dormancy and reactivation, whereas trabecular-rich marrow regions may favor initial arrest and survival due to dense sinusoidal networks and proximity to immune and stromal cells (Lawson et al., 2015). By contrast, articular cartilage is avascular and lacks marrow-type stromal niches, providing a plausible biological rationale for why hematogenous tumor cell homing is rarely centered within cartilage itself; however, direct evidence linking cartilage to a cartilage-specific “non-homing” phenotype remains limited and warrants further investigation (Sophia Fox, Bedi & Rodeo, 2009; Zhang et al., 2019).

In parallel with vascular and stromal cues, local immune surveillance is increasingly recognized as a determinant of whether disseminated PCa cells persist in dormancy or progress to overt outgrowth in bone (Gonzalez, Robles & Werb, 2018; Owen et al., 2020; Singh et al., 2021). Single-cell and functional profiling of human PCa bone metastases has highlighted exhausted/dysfunctional T-cell states together with expansion of immunosuppressive myeloid programs (including myeloid-derived suppressor cells (MDSC)- and tumor-associated macrophages (TAM)-like populations), which can blunt antitumor control and promote niche permissiveness (Kfoury et al., 2021; Palena & Gulley, 2021). Additional bone marrow–resident suppressive circuits, such as regulatory T cells and myeloid–tumor hybrid phenotypes, have also been implicated in sustaining an immunosuppressive microenvironment that supports metastatic progression (Zhao et al., 2012; Ye et al., 2023).

Systemic endocrine and treatment-related factors may further modulate skeletal tropism by reshaping both tumor-intrinsic programs and the bone microenvironment (Edlund, Sung & Chung, 2004; Bienz & Saad, 2015; Ren, Esposito & Kang, 2015). Androgen receptor signaling influences multiple metastasis-relevant pathways, and tumor-derived factors such as parathyroid hormone–related peptide (PTHrP) can promote osteogenic remodeling, potentially enhancing niche permissiveness for metastatic outgrowth (Liao et al., 2008; Aurilio et al., 2020). In addition, androgen deprivation therapy (ADT) increases bone turnover and fracture risk, which could indirectly alter niche dynamics and inflammatory signaling in bone (Planas Morin & Morote Robles, 2012; Ren, Esposito & Kang, 2015). Nonetheless, the directionality and causality of therapy-induced microenvironmental changes on metastasis initiation remain incompletely defined and likely depend on disease stage and treatment timing. Likewise, primary tumor features (*e.g*., grade/stage, vascular invasion or perineural invasion, and genomic alterations such as DNA repair defects) are associated with metastatic propensity at the patient level (Zareba et al., 2017), but how these traits mechanistically interface with specific bone niches is not fully resolved and remains an important area for future work. Finally, pre-existing bone status (*e.g*., osteoporosis, microdamage, or prior fractures) may influence remodeling intensity and local cytokine milieus, potentially affecting the availability of permissive niches (Elaasser, Arakil & Mohammad, 2024; Duong, Kafer & Maugham-Macan, 2025); However, distinguishing bone fragility as a comorbidity from true changes in metastatic incidence will require carefully controlled clinical and translational studies.

## Cellular contributors to prostate cancer bone metastasis

### Osteoclast-mediated bone resorption plays a limited role

OCLs are specialized multinucleated cells responsible for bone resorption, a process essential for skeletal homeostasis. By degrading the extracellular matrix, OCLs remove aged, damaged, or biomechanically compromised bone tissue, thereby creating space for new bone formation (Sabe, Yahara & Ishii, 2024). Within the metastatic niche, however, this physiological role is co-opted to support tumor progression. During resorption, OCLs release matrix-embedded growth factors such as TGF-β, which enhance tumor cell proliferation, survival, and metastatic potential (Prigol et al., 2023). In PCa bone lesions, heightened OCL activity is often observed at lesion margins, particularly in osteolytic or mixed lesion types, where it is associated with tumor expansion and skeletal dissemination (Archer Goode, Wang & Munkley, 2023). Beyond direct effects on tumor cells, TGF-β released during resorption participates in coupling by modulating osteoclast differentiation/resorptive activity and recruiting/activating osteoprogenitors to support subsequent osteoblast-mediated formation (Crane, Xian & Cao, 2016; Wu, Chen & Li, 2016).

Given these mechanisms, OCL inhibition has been investigated as a therapeutic strategy for PCa bone metastases. The two principal pharmacologic approaches are bisphosphonates—synthetic pyrophosphate analogs that inhibit bone resorption—and denosumab, a monoclonal antibody targeting RANKL (Wani et al., 2025). Yet, clinical outcomes in PCa have been modest. Bisphosphonate therapy failed to significantly improve overall survival or effectively prevent bone metastasis onset compared with standard care (Arjunan et al., 2023). Denosumab has shown modest gains in progression-free and overall survival in certain studies, but its clinical impact remains limited (Widyadharma et al., 2024).

Together, these findings indicate that osteoclast-targeted therapies alone exert only marginal effects on PCa progression. This suggests that other bone-resident cell types, possibly with more dominant roles than osteoclasts, are central drivers of the PCa–bone metastatic axis.

### Osteoblast-mediated niche maintenance may recruit prostate cancer cells to bone

PCa exhibits a strong predilection for metastasis to bone, particularly the bone marrow (BM), where colonization begins with tumor cell invasion of the marrow microenvironment (Jiang, 2025). To establish metastases, circulating tumor cells must extravasate through vascular endothelium and home to BM niches (Ji, Wu & Jiang, 2023). The spatial overlap between PCa lesions and hematopoietic sites suggests that the BM provides a supportive microenvironment for metastatic seeding, with chemokines and adhesion molecules playing critical roles in tumor cell retention within marrow vasculature (Agafonova et al., 2025).

Under physiological conditions, OBs regulate hematopoietic stem cell (HSC) niches *via* the CXCR4/CXCL12 axis, which controls HSC localization in BM (Gao et al., 2024; Kwon et al., 2024). PCa cells exploit this pathway: OB-derived CXCL12 binds CXCR4 receptors on tumor cells, directing their recruitment to bone (Johnson & Cook, 2023). In this process, PCa cells compete with HSCs for niche occupancy, leveraging OB signals to enhance survival (Keller, 2011). Downstream activation of mitogen-activated protein kinase (MAPK), PI3K/Akt, Ras, Rho GTPases, and NF-κB pathways promotes tumor proliferation, invasion, and inflammatory signaling (Lei et al., 2022). Notably, CXCL12 expression is enriched in epiphyseal regions, underscoring the spatial specificity of PCa homing through CXCR4/CXCL12 signaling (Archer Goode, Wang & Munkley, 2023).

Imaging studies using two-photon microscopy and histomorphometry confirm that PCa cells preferentially home to osteoblast-rich regions during early metastasis. Pharmacologic blockade of CXCR4/CXCL12 signaling with AMD3100 mobilizes PCa cells from the niche without impairing viability, highlighting the pathway’s role in skeletal colonization (Wang et al., 2014). Conversely, PTH-induced osteoblast expansion enhances PCa colonization within BM (Archer Goode, Wang & Munkley, 2023). These findings support the CXCR4/CXCL12 axis as a promising therapeutic target for limiting metastatic seeding and progression.

In addition, Annexin A2 (AnxA2)—a calcium-dependent phospholipid-binding protein expressed by OBs—plays a complementary role in PCa cell recruitment. AnxA2 is critical for HSC anchorage in the BM niche, and its dysregulation during tumor implantation disrupts niche homeostasis. Interactions between CXCR4/CXCL12 and AnxA2/CXCL12 pathways further promote PCa cell localization to the endosteal niche (Jung et al., 2015; Yao & Zhang, 2022).

Upon homing, OB–PCa interactions mediated by AnxA2 initiate two key dormancy-related responses: (i) upregulation of dormancy-associated receptors Axl, Sky, and Mer in PCa cells (Axelrod et al., 2019); and (ii) induction of osteoblast-derived GAS6 (growth arrest–specific 6), which activates TAM family tyrosine kinase receptors (MER, TYRO3, AXL) to suppress proliferation (Shiozawa et al., 2010). Contact with OBs enhances AXL expression in PCa cells, enabling GAS6–AXL signaling to enforce dormancy (Archer Goode, Wang & Munkley, 2023). This mechanism allows disseminated tumor cells to persist in a quiescent state within the bone microenvironment, supporting long-term survival during early metastatic colonization. Here, TAM refers to the TYRO3–AXL–MER receptor family, not tumor-associated macrophages.

Targeting osteoblast-mediated dormancy pathways may provide therapeutic opportunities to eradicate dormant disseminated tumor cells before reactivation and overt metastatic outgrowth.

#### Multifaceted effects of osteoblasts on prostate cancer growth

Interactions between OBs and PCa cells are multifaceted, exerting both tumor-promoting and tumor-suppressive effects depending on spatial and temporal context (Archer Goode, Wang & Munkley, 2023). OBs form gap junctions with PCa cells, enabling direct intercellular communication and calcium ion (Ca^2+^) transfer. Calcium signaling contributes to tumor proliferation and may facilitate metastatic dissemination (Ardura et al., 2020). Altered intracellular Ca^2+^ levels influence cell cycle progression, a critical driver of unchecked tumor growth (Moon, 2023). In addition, calcium dysregulation disrupts protein interactions and signaling cascades, leading to metabolic reprogramming that can enhance immune evasion and metastatic potential (Nong et al., 2023).

Despite these pro-tumor influences, OBs also provide inhibitory cues. For example, OB-derived Wnt5a suppresses PCa proliferation and metastasis *in vivo*. Interestingly, Wnt5a-treated PCa cells develop resistance to docetaxel, a frontline agent for metastatic PCa, suggesting that Wnt5a induces a dormant phenotype (Ren et al., 2019). This highlights the context-dependent nature of OB–tumor signaling, where specific cytokines and growth factors yield divergent effects.

The Wnt antagonist Dickkopf-1 (Dkk1) illustrates this dynamic regulation. Dkk1 expression is elevated during early PCa progression but downregulated as tumors advance to skeletal metastasis (Yuan et al., 2023). Functionally, Dkk1 acts as a molecular switch, shifting PCa lesions from osteolytic to osteoblastic phenotypes (Hall et al., 2008).

Similarly, mature OBs promote tumor dormancy by secreting TGF-β and growth differentiation factor 10 (GDF10) (Yu-Lee et al., 2018). In early stages, TGF-β exerts antiproliferative effects through cell cycle arrest and apoptosis. However, as disease progresses, these inhibitory effects wane. TGF-β then promotes metastasis by driving epithelial–mesenchymal transition (EMT) *via* transcriptional regulators such as Snail1/2, ZEB1/2, and HMGA2, while enhancing tumor motility through PI3K/AKT signaling (Zhang, Alexander & Wang, 2017; Ma et al., 2024).

Together, these findings underscore the dual nature of OB–tumor interactions, spanning dormancy induction to metastatic activation. Appreciating this functional plasticity may inform stage- and niche-specific therapeutic strategies for PCa bone metastases.

### The role of osteocytes in prostate cancer bone metastasis

**Figure 3:**
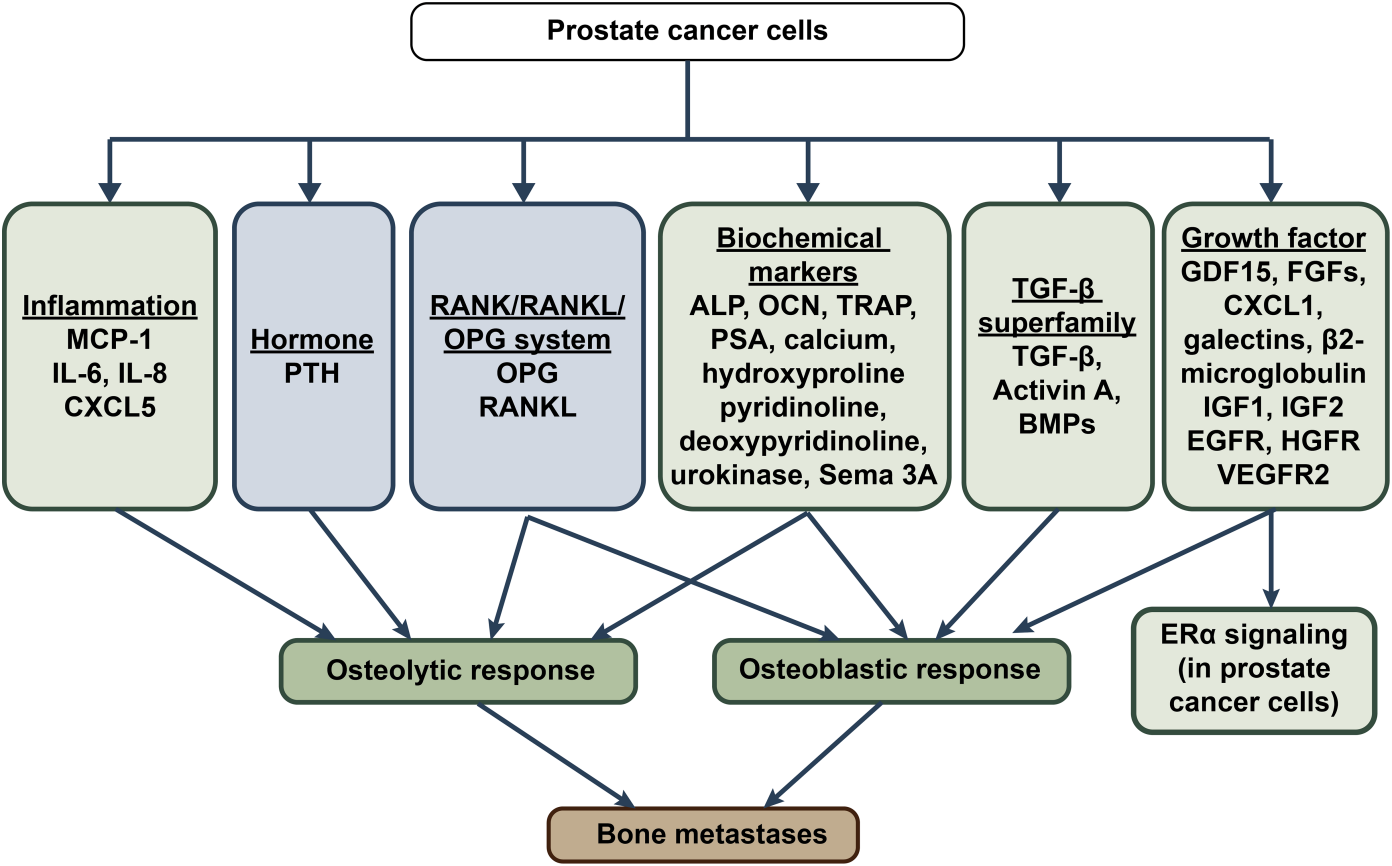
Major molecular mediators underlying osteolytic and osteoblastic responses in prostate cancer bone metastasis. Prostate cancer cells regulate bone metastasis through multiple classes of mediators, including inflammatory factors (MCP-1, IL-6, IL-8, and CCL5), hormone-related signalling (PTHrP), the RANK/RANKL/OPG axis, biochemical markers (ALP, OCN, TRAP, PSA, calcium, hydroxyproline, pyridinoline, deoxypyridinoline, urokinase, and Sema3A), the TGF-β superfamily (TGF-β, Activin A, and BMPs), and growth factor pathways (GDF15, FGFs, CXCL1, galectins, β2-microglobulin, IGF-1, IGF-2, EGFR, HGFR, and VEGFR2). These signals promote osteolytic and/or osteoblastic responses, with androgen receptor signalling further enhancing osteoblastic activity, thereby contributing to the establishment and progression of bone metastases.

Osteocytes are central regulators of bone remodeling, modulating osteoblast and osteoclast activity through secretion of receptor activator of nuclear factor-κB ligand (RANKL), sclerostin (SOST), and Dickkopf-1 (DKK1). These mediators maintain calcium homeostasis and control bone turnover (Wu et al., 2024). Acting as mechanosensors, osteocytes also coordinate skeletal responses to mechanical load and regulate phosphate metabolism (Zhao et al., 2022). Figure 3 summarizes key molecular pathways involved in prostate cancer bone metastasis.

Emerging evidence implicates osteocyte-derived factors in PCa progression. Molecules such as CCL5 and matrix metalloproteinases (MMPs) enhance PCa cell invasion *in vitro* (Guan et al., 2024). In three-dimensional bone culture models, osteocytes exposed to tumor cells display altered secretion profiles, with increased fibroblast growth factor 23 (FGF-23), RANKL, and DKK1, coupled with marked downregulation of SOST expression. These changes may facilitate the osteoblastic lesions characteristic of PCa bone metastases (Choudhary et al., 2018; Wang et al., 2020).

Interestingly, while local osteocyte-derived SOST is suppressed in tumor-associated bone, clinical studies report elevated circulating SOST levels in patients with osteoblastic PCa metastases. This apparent paradox highlights the need for further mechanistic studies to clarify the dual role of SOST in tumor–bone interactions (Bock et al., 2019).

In addition to regulatory proteins, bone-derived growth factors such as insulin-like growth factor II (IGF-II) and platelet-derived growth factor (PDGF) promote PCa cell survival and proliferation by activating the Akt/PKB pathway. Notably, PCa cells localized in bone exhibit elevated PDGF receptor expression, indicating niche-specific adaptation to bone-derived signals (Dolloff et al., 2005; Russell et al., 2010; Elemam et al., 2024).

Collectively, these findings underscore the multifaceted role of osteocytes in prostate cancer colonization, survival, and expansion within the bone microenvironment. The osteocyte–tumor axis represents a dynamic regulatory network and an emerging therapeutic target in metastatic PCa.

## Influence of prostate cancer cells on bone remodeling cells

Although the skeleton has traditionally been considered a hostile environment for tumor growth, recent evidence demonstrates that primary PCa tumors can “educate” bone marrow cells to precondition distant skeletal niches, thereby facilitating metastatic colonization (Jiang, 2025; Dai et al., 2019). This reprogramming fosters a tumor-permissive microenvironment that supports invasion, immune evasion, and metastatic progression.

Extracellular vesicles (EVs) secreted by PCa cells play a central role in this process. EVs reprogram OBs toward tumor-supportive phenotypes, thereby establishing favorable conditions for metastatic seeding and expansion (Li & Wang, 2021). For example, EVs derived from PC3 and DU-145 cell lines deliver ETV1 to murine osteoprogenitor MC3T3 cells, driving their differentiation into functional osteoblasts (educated osteoblasts, EOs) (Vlaeminck-Guillem, 2018). PC3-derived EVs also contain RNA species that activate EO signaling pathways, enhancing their capacity to support tumor growth (Probert et al., 2019). These “educated” osteoblasts internalize EVs and initiate endogenous molecular cascades that sustain PCa colonization and survival within the bone microenvironment (Brown et al., 2024).

At the endosteal surface, bone-lining cells respond to tumor cell arrival and activation (Lawson et al., 2015). OCL differentiation is stimulated through RANKL secretion by stromal and tumor cells (Chawla et al., 2024), a mechanism co-opted by PCa cells to promote osteoclastogenesis and bone resorption.

Following skeletal colonization, PCa cells preferentially accumulate in osteoblast-rich regions (Wang et al., 2014). Direct interactions with OBs can convert normal osteoblasts into cancer-associated osteoblasts (CAOs) (Kimura et al., 2017). These CAOs secrete cytokines and growth factors that further promote PCa cell homing, invasion, and metastatic expansion (Wang et al., 2019). In this context, CAOs denote osteoblast-lineage cells as defined in the cited studies (*e.g*., osteogenic-marker–positive, bone-lining populations), rather than cancer-associated fibroblasts (CAFs). Collectively, these findings highlight the essential contribution of bone remodeling and tumor-driven niche reprogramming to the establishment of PCa skeletal metastases.

### Crosstalk between prostate cancer cells and bone remodeling cells

Direct physical interactions among PCa cells, OBs, and OCLs establish a self-reinforcing growth circuit (Özdemir et al., 2014). PCa cells stimulate osteoblastic lesion development by promoting OB proliferation and differentiation (Archer Goode, Wang & Munkley, 2023). Upon colonization of bone, tumor cells secrete bioactive molecules such as TGF-β and endothelin-1, which remodel the niche to favor tumor growth (Ruksha & Palkina, 2025). These signals enhance OB activity and induce the release of insulin-like growth factor-1 (IGF-1), interleukin-6 (IL-6), and interleukin-8 (IL-8), all of which support tumor proliferation and survival (Martiniakova et al., 2023).

At the same time, tumor-induced osteoblast activation upregulates RANKL expression, stimulates parathyroid hormone–related peptide (PTHrP) signaling, and drives osteoclast activation. The resulting imbalance in bone remodeling promotes abnormal new bone deposition while simultaneously fueling tumor expansion (Satcher & Zhang, 2022; Xu et al., 2023).

**Figure 4:**
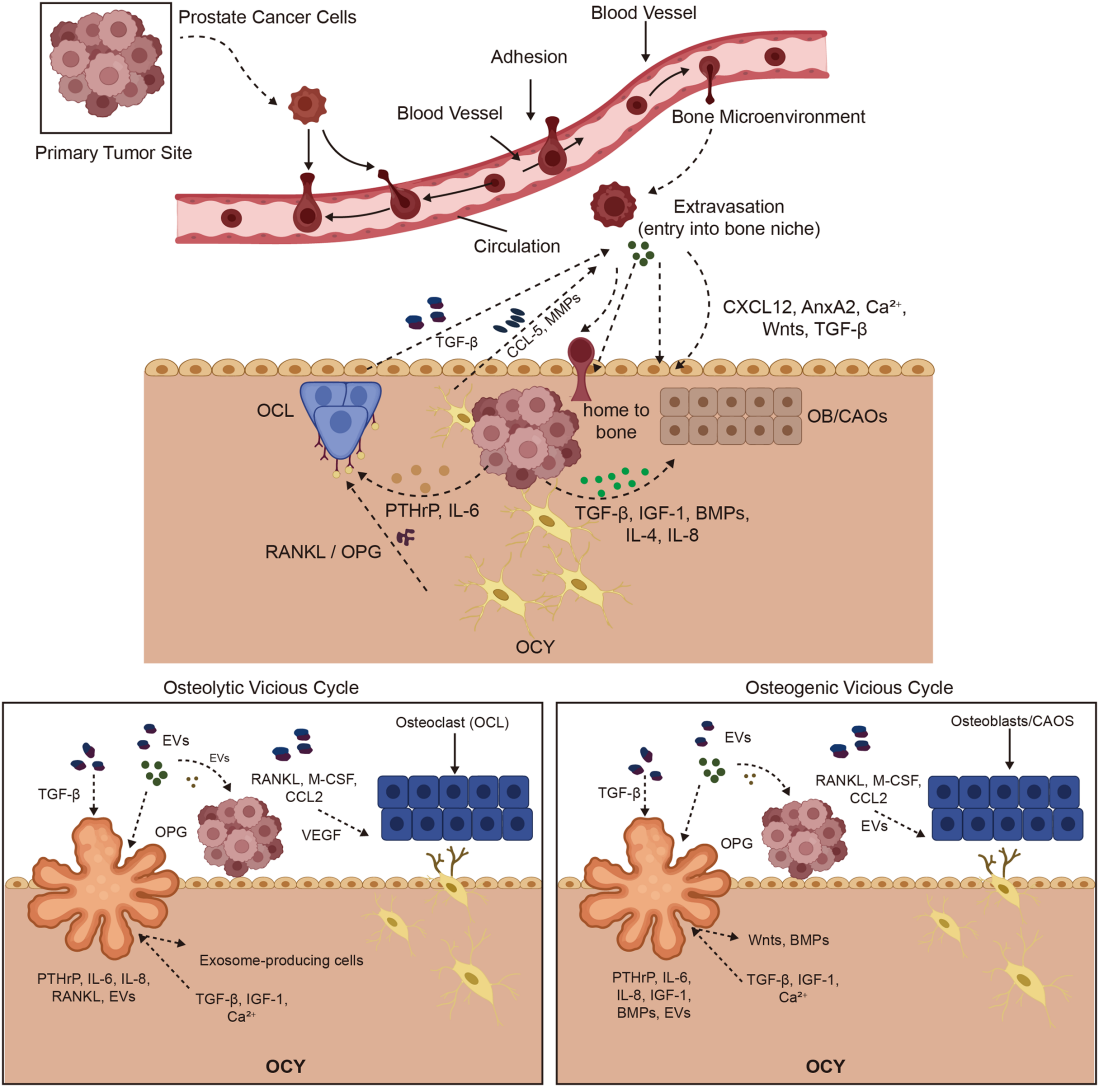
Signaling pathways involved in prostate cancer bone metastasis and bone remodeling. Schematic overview of prostate cancer bone metastasis and distinct vicious cycles within the bone microenvironment. Prostate cancer cells disseminate from the primary tumor site into the circulation, adhere to vascular endothelium, and subsequently extravasate into the bone niche. Within the bone microenvironment, tumor cells home to bone and interact with key resident cell populations, including osteoclasts (OCL), osteoblasts/cancer-associated osteoblasts (OB/CAOs), and osteocytes (OCY). Tumor- and microenvironment-derived extracellular vesicles (EVs) and soluble mediators (*e.g*., CXCL12, AnxA2, Ca^2+^, Wnts, TGF-β) facilitate colonization and reprogram stromal signaling. The lower panels depict two complementary pathogenic feedback loops: an osteolytic vicious cycle characterized by enhanced osteoclastogenesis and bone resorption (driven by factors such as PTHrP, IL-6/IL-8, RANKL, M-CSF, CCL2 and VEGF), and an osteogenic/osteoblastic vicious cycle featuring aberrant osteoblast/CAO activation (supported by Wnts and BMPs). In both settings, bone matrix degradation and remodeling release growth factors (notably TGF-β and IGF-1) and Ca^2+^, which further promote tumor growth and sustain metastatic progression.

Together, these reciprocal interactions between PCa cells and bone-resident cells establish a vicious cycle of osteogenic and osteolytic signaling. Figure 4 illustrates the osteolytic and osteogenic vicious cycles in prostate cancer bone metastasis. This cycle underpins tumor persistence within the skeleton and accelerates metastatic progression.

### Functional reprogramming of bone remodeling cells by prostate cancer cells

PCa cells actively disrupt normal bone remodeling by modulating both osteoclastic and osteoblastic activity. Upregulation of osteolytic factors such as receptor activator of nuclear factor-κB ligand (RANKL) promotes osteoclast differentiation and bone resorption, accelerating the release of matrix-embedded growth factors including TGF-β and insulin-like growth factor-1 (IGF-1), which further enhance tumor proliferation (De Leon-Oliva et al., 2023).

Concurrently, PCa cells secrete PTHrP, which stimulates osteoblast proliferation and differentiation, sustaining aberrant bone formation during tumor expansion (Liao et al., 2008). By simultaneously driving osteoblast activity and osteoclast-mediated degradation, PCa cells uncouple the physiological balance of bone remodeling (Huang et al., 2022). Growth factors released from resorbed bone reinforce tumor growth and establish a feed-forward loop that accelerates skeletal destruction and metastatic progression (Arakil et al., 2024).

### Autocrine and paracrine mechanisms of PCa cells in bone metastasis

PCa cells modulate both tumor and stromal behavior through autocrine and paracrine signals that enhance proliferation and osteogenic activity during bone colonization (Lin, Yu-Lee & Lin, 2018). Androgen receptor–driven expression of Wnt7b supports PCa growth while simultaneously promoting osteoblast differentiation through direct tumor–osteoblast interactions (Zheng et al., 2013). Elevated Wnt7b expression in osteoblastic lesions reinforces the concept of osteomimicry, whereby PCa cells acquire bone-like properties that enable survival and expansion within the skeletal niche (Yu et al., 2024).

Core-binding factor alpha 1 (Cbfa1/Runx2) is a key transcriptional regulator of this adaptation. Runx2 promotes tumor progression by activating both TGF-β and androgen receptor signaling pathways (van der Deen et al., 2010; Shuai & Huang, 2025). In addition, Runx2-driven production of PTHrP can be proteolytically cleaved by prostate-specific antigen (PSA), shifting the bone response toward osteogenesis by reducing resorption (Datta & Abou-Samra, 2009; Zhu et al., 2024).

Together, these findings highlight how PCa cells leverage osteomimicry and Runx2-mediated signaling to remodel the bone niche and sustain metastatic growth.

### Tumor-derived factors promote osteogenesis and angiogenesis

Tumor-derived PTHrP not only stimulates vascular endothelial growth factor (VEGF) production *via* autocrine signaling but also promotes osteoblast-mediated bone formation and angiogenesis, thereby supporting tumor expansion within the skeleton^96^. VEGF serves as a critical regulator of osteoblast–osteoclast crosstalk in the bone marrow (Wang et al., 2025a). In conditioned media from metastatic C4-2B PCa cells, VEGF enhances osteoblast mineralization, an effect attenuated by VEGF-neutralizing antibodies or the bone morphogenetic protein (BMP) inhibitor noggin, indicating a cooperative role of tumor-derived VEGF and BMPs in osteogenic activation (Dai et al., 2004). In addition, tumor-derived endothelin-1 (ET-1) and ETA receptor signaling have been implicated in osteoblastic responses in PCa bone metastasis (Guise, Yin & Mohammad, 2003).

Although VEGF alone can induce osteoblast differentiation, it is insufficient to drive mineralization in the absence of additional tumor-derived factors, suggesting that PCa-induced osteogenesis is governed by a multifactorial network (Kitagawa et al., 2005).

Collectively, these findings illustrate how PCa cells hijack bone remodeling and vascular signaling pathways to establish and sustain skeletal metastases.

### Prostate cancer–osteocyte interactions and their role in dysregulated bone remodeling

Recent studies highlight the pivotal role of OCYs in the pathogenesis of PCa bone metastases. As the most abundant and longest-lived skeletal cells (Anloague & Delgado-Calle, 2023), OCYs are strategically positioned to interact with disseminated PCa cells during bone colonization. In immunocompetent mouse models, intratibial injection of PCa cells demonstrates direct tumor–osteocyte contact, accompanied by enlarged lacunae and impaired mechanosensory function of the bone network (Verbruggen, 2024).

These structural alterations are especially pronounced in osteosclerotic lesions, where expansion of lacunar volume coincides with reduced canalicular density, reflecting compromised osteocytic signaling and diminished remodeling capacity (Tiede-Lewis & Dallas, 2019). By disrupting the lacunocanalicular system, PCa cells blunt OCY responsiveness to mechanical cues, thereby contributing to the imbalance in bone turnover characteristic of metastatic progression.

*In vitro* studies further reveal that mechanical pressure exerted by tumor growth dysregulates OCY function, triggering release of pro-tumorigenic mediators such as chemokine CCL5 and MMPs (Sottnik et al., 2015). CCL5 acts as a chemoattractant for immune cells, while MMPs degrade extracellular matrix, facilitating invasion and tissue remodeling (Yu-Ju Wu et al., 2020). Together, these factors enhance the motility and invasiveness of PCa cell lines. Consistently, CCL5 has been implicated in metastatic promotion across multiple malignancies (Huang et al., 2020). Table 1 summarizes key signaling pathways involved in prostate cancer (PCa) bone metastasis and their interaction with bone remodeling cells.

**Table 1:** Key signaling pathways of bone remodeling cells in prostate cancer bone metastasis and current research challenges.

Key signaling pathways	Bone remodeling cells involved	Role in prostate cancer bone metastasis	Research challenges	References
Wnt5a-mediated dormancy	OBs	Regulates tumor dormancy, suppresses proliferation, and induces resistance to chemotherapy.	Further research is needed to understand the specific mechanisms of Wnt5a in different environments and its regulation of the balance between dormancy and proliferation.	Ren et al. (2019), Zarrer & Taipaleenmäki (2024)
TGF-β–induced epithelial-mesenchymal transition (EMT)	OBs	Induces epithelial-mesenchymal transition (EMT), promoting metastasis in later stages of cancer.	Overcoming the resistance to TGF-β pathway inhibitors in treatment and clarifying the role of TGF-β in different stages of metastatic progression.	Zhang, Alexander & Wang (2017), Ma et al. (2024)
CXCL12/CXCR4 axis	OBs, HSCs	Regulates recruitment of prostate cancer cells to bone marrow for metastasis.	Developing more effective strategies to inhibit the CXCL12/CXCR4 signaling pathway to overcome the complexity of metastatic spread.	Keller (2011), Zarrer & Taipaleenmäki (2024)
RANKL/OPG axis	OCLs, OBs	Regulates osteoclast differentiation and bone resorption, promoting tumor progression.	Although RANKL inhibition shows some clinical benefits, its efficacy remains limited, requiring further investigation into its mechanisms and the optimization of therapeutic strategies.	Elaasser, Arakil & Mohammad (2024), Wani et al. (2025)
SOST/Dkk1/WNT pathway	OCYs, OBs	Regulates osteoblast differentiation and bone formation in response to mechanical cues.	The dual role of SOST in the tumor-bone microenvironment is not fully understood, and further research is needed to clarify its specific mechanisms in metastasis.	Galea, Lanyon & Price (2017), Yuze et al. (2025)
VEGF signaling	OBs, OCLs	Regulates angiogenesis and bone formation, promoting prostate cancer expansion in bone.	The interaction between VEGF and other tumor-derived factors in osteogenesis is complex, and targeting these pathways simultaneously remains a challenge.	Dai et al. (2004), Kitagawa et al. (2005)

These findings underscore the underappreciated role of osteocytes in tumor–bone interactions. By co-opting the osteocytic network, PCa cells evade mechanical surveillance, reshape the bone microenvironment, and promote pathologic remodeling. Elucidating the bidirectional signaling between OCYs and PCa cells may provide novel therapeutic opportunities to disrupt the vicious cycle of bone metastasis.

## Conclusion

In summary, this review delineates the multifaceted roles of osteoblasts, osteoclasts, and osteocytes in the initiation and progression of PCa bone metastases. Particular emphasis is placed on the dynamic, reciprocal interactions between disseminated tumor cells and the bone microenvironment, illustrating how PCa cells exploit physiological remodeling pathways to promote colonization, immune evasion, dormancy, and subsequent proliferation. Key signaling networks—including CXCL12/CXCR4, OPG/RANKL, Wnt, and TGF-β—as well as tumor-derived mediators such as PTHrP, Runx2, and VEGF, collectively shape osteogenic and osteolytic responses that sustain metastatic growth.

Despite substantial advances, the molecular events governing phenotypic switching, metastatic niche formation, and tumor–bone co-evolution remain incompletely defined. Future research integrating high-resolution imaging, single-cell transcriptomics, and physiologically relevant *in vivo* models will be essential to unravel these mechanisms. Such insights hold promise for the development of more effective bone-targeted therapies and improved clinical management of metastatic PCa. Future work should explicitly address how marrow vascular niches and immune states regulate dormancy *vs* reactivation, how systemic therapies reshape these niches, and which tumor-intrinsic features predict bone-tropic dissemination.

## Additional Information and Declarations

### Data Availability

The following information was supplied regarding data availability:

This is a Literature Review.

## References

[ref-1] Adejuyigbe B, Kallini J, Chiou D, Kallini JR (2023). Osteoporosis: molecular pathology, diagnostics, and therapeutics. International Journal of Molecular Sciences.

[ref-2] Agafonova A, Prinzi C, Trovato Salinaro A, Ledda C, Cosentino A, Cambria MT, Anfuso CD, Lupo G (2025). Breaking barriers: the role of the bone marrow microenvironment in multiple myeloma progression. International Journal of Molecular Sciences.

[ref-3] Anloague A, Delgado-Calle J (2023). Osteocytes: new kids on the block for cancer in bone therapy. Cancers.

[ref-4] Arakil N, Akhund SA, Elaasser B, Mohammad KS (2024). Intersecting paths: unraveling the complex journey of cancer to bone metastasis. Biomedicines.

[ref-5] Archer Goode E, Wang N, Munkley J (2023). Prostate cancer bone metastases biology and clinical management (review). Oncology Letters.

[ref-6] Ardura JA, Álvarez-Carrión L, Gutiérrez-Rojas I, Alonso V (2020). Role of calcium signaling in prostate cancer progression: effects on cancer hallmarks and bone metastatic mechanisms. Cancers.

[ref-7] Arjunan D, Bhadada T, Mohankumar SB, Bhadada SK (2023). Non-biological antiresorptive: bisphosphonates. Indian Journal of Orthopaedics.

[ref-8] Aurilio G, Cimadamore A, Mazzucchelli R, Lopez-Beltran A, Verri E, Scarpelli M, Massari F, Cheng L, Santoni M, Montironi R (2020). Androgen receptor signaling pathway in prostate cancer: from genetics to clinical applications. Cells.

[ref-9] Axelrod HD, Valkenburg KC, Amend SR, Hicks JL, Parsana P, Torga G, DeMarzo AM, Pienta KJ (2019). AXL is a putative tumor suppressor and dormancy regulator in prostate cancer. Molecular Cancer Research.

[ref-10] Ayzman A, Pachynski RK, Reimers MA (2025). PSMA-based therapies and novel therapies in advanced prostate cancer: the now and the future. Current Treatment Options in Oncology.

[ref-11] Baldessari C, Pipitone S, Molinaro E, Cerma K, Fanelli M, Nasso C, Oltrecolli M, Pirola M, D’Agostino E, Pugliese G, Cerri S, Vitale MG, Madeo B, Dominici M, Sabbatini R (2023). Bone metastases and health in prostate cancer: from pathophysiology to clinical implications. Cancers.

[ref-12] Batoon L, McCauley LK (2021). Cross talk between macrophages and cancer cells in the bone metastatic environment. Frontiers in Endocrinology.

[ref-13] Bienz M, Saad F (2015). Androgen-deprivation therapy and bone loss in prostate cancer patients: a clinical review. Bonekey Reports.

[ref-14] Bock N, Shokoohmand A, Kryza T, Röhl J, Meijer J, Tran PA, Nelson CC, Clements JA, Hutmacher DW (2019). Engineering osteoblastic metastases to delineate the adaptive response of androgen-deprived prostate cancer in the bone metastatic microenvironment. Bone Research.

[ref-15] Brown TJ, Rutland CS, Choi KK, Tse F, Peffers MJ, Mongan NP, Arkill KP, Ritchie A, Clarke PA, Ratan H, Allegrucci C, Grabowska AM, James V (2024). Modulation of the pre-metastatic bone niche: molecular changes mediated by bone-homing prostate cancer extracellular vesicles. Frontiers in Cell and Developmental Biology.

[ref-16] Buijs JT, Stayrook KR, Guise TA (2011). TGF-β in the bone microenvironment: role in breast cancer metastases. Cancer Microenvironment: Official Journal of the International Cancer Microenvironment Society.

[ref-17] Cao Z, Zheng R, Li J, Wang X, Ding C, Zhang F, Geng J, Wei Z, Fan R (2025). Risk factors of bone metastasis in lung adenocarcinoma. BMC Pulmonary Medicine.

[ref-18] Chawla P, Sharma I, Gau D, Eder I, Chen F, Yu V, Welling N, Boone D, Taboas J, Lee AV, Larregina A, Galson DL, Roy P (2024). Breast cancer cells promote osteoclast differentiation in an MRTF-dependent paracrine manner. Molecular Biology of the Cell.

[ref-19] Chen F, Han Y, Kang Y (2021). Bone marrow niches in the regulation of bone metastasis. British Journal of Cancer.

[ref-20] Chen Y, Jia L, Han T, Zhao Z, Yang J, Xiao J, Yang H-J, Yang K (2024). Osteoporosis treatment: current drugs and future developments. Frontiers in Pharmacology.

[ref-21] Choi IA, Umemoto A, Mizuno M, Park-Min K-H (2024). Bone metabolism—an underappreciated player. npj Metabolic Health and Disease.

[ref-22] Choudhary S, Ramasundaram P, Dziopa E, Mannion C, Kissin Y, Tricoli L, Albanese C, Lee W, Zilberberg J (2018). Human ex vivo 3D bone model recapitulates osteocyte response to metastatic prostate cancer. Scientific Reports.

[ref-23] Crane JL, Xian L, Cao X (2016). Role of TGF-β signaling in coupling bone remodeling. Methods in Molecular Biology.

[ref-24] Dai J, Escara-Wilke J, Keller JM, Jung Y, Taichman RS, Pienta KJ, Keller ET (2019). Primary prostate cancer educates bone stroma through exosomal pyruvate kinase M2 to promote bone metastasis. Journal of Experimental Medicine.

[ref-25] Dai J, Kitagawa Y, Zhang J, Yao Z, Mizokami A, Cheng S, Nör J, McCauley LK, Taichman RS, Keller ET (2004). Vascular endothelial growth factor contributes to the prostate cancer-induced osteoblast differentiation mediated by bone morphogenetic protein. Cancer Research.

[ref-26] Daponte V, Henke K, Drissi H (2024). Current perspectives on the multiple roles of osteoclasts: mechanisms of osteoclast-osteoblast communication and potential clinical implications. eLife.

[ref-27] Datta NS, Abou-Samra AB (2009). PTH and PTHrP signaling in osteoblasts. Cellular Signalling.

[ref-28] De Leon-Oliva D, Barrena-Blázquez S, Jiménez-Álvarez L, Fraile-Martinez O, García-Montero C, López-González L, Torres-Carranza D, García-Puente LM, Carranza ST, MÁ Á-M, Álvarez-Mon M, Diaz R, Ortega MA (2023). The RANK-RANKL–OPG system: a multifaceted regulator of homeostasis, immunity, and cancer. Medicina.

[ref-29] Dolloff NG, Shulby SS, Nelson AV, Stearns ME, Johannes GJ, Thomas JD, Meucci O, Fatatis A (2005). Bone-metastatic potential of human prostate cancer cells correlates with Akt/PKB activation by α platelet-derived growth factor receptor. Oncogene.

[ref-30] Duong HQ, Kafer G, Maugham-Macan M (2025). Bone metastasis in endocrine-related cancer: unravelling invasion and destruction. Endocrine-Related Cancer.

[ref-31] Edlund M, Sung S-Y, Chung LWK (2004). Modulation of prostate cancer growth in bone microenvironments. Journal of Cellular Biochemistry.

[ref-32] Elaasser B, Arakil N, Mohammad KS (2024). Bridging the gap in understanding bone metastasis: a multifaceted perspective. International Journal of Molecular Sciences.

[ref-33] Elemam NM, Hotait HY, Saleh MA, El-Huneidi W, Talaat IM (2024). Insulin-like growth factor family and prostate cancer: new insights and emerging opportunities. Frontiers in Endocrinology.

[ref-34] Galea GL, Lanyon LE, Price JS (2017). Sclerostin’s role in bone’s adaptive response to mechanical loading. Bone.

[ref-35] Gao L, Lee H, Goodman JH, Ding L (2024). Hematopoietic stem cell niche generation and maintenance are distinguishable by an epitranscriptomic program. Cell.

[ref-36] Gonzalez H, Robles I, Werb Z (2018). Innate and acquired immune surveillance in the postdissemination phase of metastasis. FEBS Journal.

[ref-37] Guan Y, Liu X, Tian J, Yang G, Xu F, Guo N, Guo L, Wan Z, Huang Z, Gao M, Chong T (2024). CCL5 promotes the epithelial-mesenchymal transition of circulating tumor cells in renal cancer. Journal of Translational Medicine.

[ref-38] Guise TA, Yin JJ, Mohammad KS (2003). Role of endothelin-1 in osteoblastic bone metastases. Cancer.

[ref-39] Hall CL, Daignault SD, Shah RB, Pienta KJ, Keller ET (2008). Dickkopf-1 expression increases early in prostate cancer development and decreases during progression from primary tumor to metastasis. The Prostate.

[ref-40] Hansdah K, Lui JC (2024). Emerging insights into the endocrine regulation of bone homeostasis by gut microbiome. Journal of the Endocrine Society.

[ref-41] Harsini S, Wilson D, Saprunoff H, Allan H, Gleave M, Goldenberg L, Chi KN, Kim-Sing C, Tyldesley S, Bénard F (2023). Outcome of patients with biochemical recurrence of prostate cancer after PSMA PET/CT-directed radiotherapy or surgery without systemic therapy. Cancer Imaging.

[ref-42] Hu H-T, Zhang Z-Y, Luo Z-X, Ti H-B, Wu J-J, Nie H, Yuan Z-D, Wu X, Zhang K-Y, Shi S-W, Qian Y-Q, Wang X-C, Wu J-J, Li X, Yuan F-L (2025). Emerging regulated cell death mechanisms in bone remodeling: decoding ferroptosis, cuproptosis, disulfidptosis, and PANoptosis as therapeutic targets for skeletal disorders. Cell Death Discovery.

[ref-43] Huang J, Freyhult E, Buckland R, Josefsson A, Damber J-E, Welén K (2022). Osteoclasts directly influence castration-resistant prostate cancer cells. Clinical & Experimental Metastasis.

[ref-44] Huang R, Wang S, Wang N, Zheng Y, Zhou J, Yang B, Wang X, Zhang J, Guo L, Wang S, Chen Z, Wang Z, Xiang S (2020). CCL5 derived from tumor-associated macrophages promotes prostate cancer stem cells and metastasis via activating β-catenin/STAT3 signaling. Cell Death and Disease.

[ref-46] Ji S, Wu W, Jiang Q (2023). Crosstalk between endothelial cells and tumor cells: a new era in prostate cancer progression. International Journal of Molecular Sciences.

[ref-47] Jiang H (2025). Prostate cancer bone metastasis: molecular mechanisms of tumor and bone microenvironment. Cancer Management and Research.

[ref-48] Johnson CS, Cook LM (2023). Osteoid cell-derived chemokines drive bone-metastatic prostate cancer. Frontiers in Oncology.

[ref-49] Jung Y, Wang J, Lee E, McGee S, Berry JE, Yumoto K, Dai J, Keller ET, Shiozawa Y, Taichman RS (2015). Annexin 2-CXCL12 interactions regulate metastatic cell targeting and growth in the bone marrow. Molecular Cancer Research.

[ref-50] Keller ET (2011). Prostate cancer cells metastasize to the hematopoietic stem cell niche in bone. Asian Journal of Andrology.

[ref-51] Kfoury Y, Baryawno N, Severe N, Mei S, Gustafsson K, Hirz T, Brouse T, Scadden EW, Igolkina AA, Kokkaliaris K, Choi BD, Barkas N, Randolph MA, Shin JH, Saylor PJ, Scadden DT, Sykes DB, Kharchenko PV, as part of the Boston Bone Metastases Consortium (2021). Human prostate cancer bone metastases have an actionable immunosuppressive microenvironment. Cancer Cell.

[ref-52] Kim JH, Seong S, Kim K, Kim I, Park JW, Koh J-T, Kim N (2025). Rac1-dependent regulation of osteoclast and osteoblast differentiation by developmentally regulated GTP-binding 2. Cell Death Discovery.

[ref-53] Kimura Y, Matsugaki A, Sekita A, Nakano T (2017). Alteration of osteoblast arrangement via direct attack by cancer cells: new insights into bone metastasis. Scientific Reports.

[ref-54] Kitagawa Y, Dai J, Zhang J, Keller JM, Nor J, Yao Z, Keller ET (2005). Vascular endothelial growth factor contributes to prostate cancer-mediated osteoblastic activity. Cancer Research.

[ref-55] Kusumbe AP (2016). Vascular niches for disseminated tumour cells in bone. Journal of Bone Oncology.

[ref-56] Kwon M, Kim BS, Yoon S, Oh S-O, Lee D (2024). Hematopoietic stem cells and their niche in bone marrow. International Journal of Molecular Sciences.

[ref-57] Lawson MA, McDonald MM, Kovacic N, Hua Khoo W, Terry RL, Down J, Kaplan W, Paton-Hough J, Fellows C, Pettitt JA, Neil Dear T, Van Valckenborgh E, Baldock PA, Rogers MJ, Eaton CL, Vanderkerken K, Pettit AR, Quinn JMW, Zannettino ACW, Phan TG, Croucher PI (2015). Osteoclasts control reactivation of dormant myeloma cells by remodelling the endosteal niche. Nature Communications.

[ref-58] Le TK, Duong QH, Baylot V, Fargette C, Baboudjian M, Colleaux L, Taïeb D, Rocchi P (2023). Castration-resistant prostate cancer: from uncovered resistance mechanisms to current treatments. Cancers.

[ref-59] Lei Z-N, Teng Q-X, Tian Q, Chen W, Xie Y, Wu K, Zeng Q, Zeng L, Pan Y, Chen Z-S, He Y (2022). Signaling pathways and therapeutic interventions in gastric cancer. Signal Transduction and Targeted Therapy.

[ref-60] Li S, Wang W (2021). Extracellular vesicles in tumors: a potential mediator of bone metastasis. Frontiers in Cell and Developmental Biology.

[ref-61] Liao J, Li X, Koh AJ, Berry JE, Thudi N, Rosol TJ, Pienta KJ, McCauley LK (2008). Tumor expressed PTHrP facilitates prostate cancer-induced osteoblastic lesions. International Journal of Cancer. Journal International Du Cancer.

[ref-62] Lin S-C, Yu-Lee L-Y, Lin S-H (2018). Osteoblastic factors in prostate cancer bone metastasis. Current Osteoporosis Reports.

[ref-63] Ma Q, Ye S, Liu H, Zhao Y, Mao Y, Zhang W (2024). HMGA2 promotes cancer metastasis by regulating epithelial-mesenchymal transition. Frontiers in Oncology.

[ref-64] Marahleh A, Kitaura H, Ohori F, Noguchi T, Mizoguchi I (2023). The osteocyte and its osteoclastogenic potential. Frontiers in Endocrinology.

[ref-65] Martin TJ, Seeman E (2023). Bone remodeling and modeling: cellular targets for antiresorptive and anabolic treatments, including approaches through the parathyroid hormone (PTH)/PTH-related protein pathway. Neurospine.

[ref-66] Martiniakova M, Mondockova V, Biro R, Kovacova V, Babikova M, Zemanova N, Ciernikova S, Omelka R (2023). The link between bone-derived factors osteocalcin, fibroblast growth factor 23, sclerostin, lipocalin 2 and tumor bone metastasis. Frontiers in Endocrinology.

[ref-67] McDonald MM, Kim AS, Mulholland BS, Rauner M (2021). New insights into osteoclast biology. JBMR Plus.

[ref-68] Moon D-O (2023). Calcium’s role in orchestrating cancer apoptosis: mitochondrial-centric perspective. International Journal of Molecular Sciences.

[ref-69] Moura SR, Olesen JB, Lindberg-Larsen M, Barbosa MA, Søe K, Almeida MI (2025). Non-coding RNA modulation in osteoclasts and its implications for osteoblast lineage cell behavior in a co-culture system. Cell Communication and Signaling.

[ref-70] Nong S, Han X, Xiang Y, Qian Y, Wei Y, Zhang T, Tian K, Shen K, Yang J, Ma X (2023). Metabolic reprogramming in cancer: mechanisms and therapeutics. MedComm.

[ref-71] Owen KL, Gearing LJ, Zanker DJ, Brockwell NK, Khoo WH, Roden DL, Cmero M, Mangiola S, Hong MK, Spurling AJ, McDonald M, Chan C-L, Pasam A, Lyons RJ, Duivenvoorden HM, Ryan A, Butler LM, Mariadason JM, Giang Phan T, Hayes VM, Sandhu S, Swarbrick A, Corcoran NM, Hertzog PJ, Croucher PI, Hovens C, Parker BS (2020). Prostate cancer cell-intrinsic interferon signaling regulates dormancy and metastatic outgrowth in bone. EMBO Reports.

[ref-72] Özdemir BC, Hensel J, Secondini C, Wetterwald A, Schwaninger R, Fleischmann A, Raffelsberger W, Poch O, Delorenzi M, Temanni R, Mills IG, van der PG, Thalmann GN, Cecchini MG (2014). The molecular signature of the stroma response in prostate cancer-induced osteoblastic bone metastasis highlights expansion of hematopoietic and prostate epithelial stem cell niches. PLOS ONE.

[ref-73] Palena C, Gulley JL (2021). A rare insight into the immunosuppressive landscape of prostate cancer bone metastases. Cancer Cell.

[ref-74] Planas Morin J, Morote Robles J (2012). Skeletal complications of ADT: disease burden and treatment options. Asian Journal of Andrology.

[ref-75] Prigol AN, Rode MP, da Luz Efe F, Saleh NA, Creczynski-Pasa TB (2023). The bone microenvironment soil in prostate cancer metastasis: an miRNA approach. Cancers.

[ref-76] Probert C, Dottorini T, Speakman A, Hunt S, Nafee T, Fazeli A, Wood S, Brown JE, James V (2019). Communication of prostate cancer cells with bone cells via extracellular vesicle RNA; a potential mechanism of metastasis. Oncogene.

[ref-77] Ren D, Dai Y, Yang Q, Zhang X, Guo W, Ye L, Huang S, Chen X, Lai Y, Du H, Lin C, Peng X, Song L (2019). Wnt5a induces and maintains prostate cancer cells dormancy in bone. Journal of Experimental Medicine.

[ref-78] Ren G, Esposito M, Kang Y (2015). Bone metastasis and the metastatic niche. Journal of Molecular Medicine.

[ref-79] Ruksha T, Palkina N (2025). Role of exosomes in transforming growth factor-β-mediated cancer cell plasticity and drug resistance. Exploration of Targeted Anti-Tumor Therapy.

[ref-80] Russell MR, Liu Q, Lei H, Kazlauskas A, Fatatis A (2010). The α-receptor for platelet derived growth factor confers bone-metastatic potential to prostate cancer cells by ligand- and dimerization-independent mechanisms. Cancer Research.

[ref-81] Sabe H, Yahara Y, Ishii M (2024). Cell fusion dynamics: mechanisms of multinucleation in osteoclasts and macrophages. Inflammation and Regeneration.

[ref-82] Satcher RL, Zhang XH-F (2022). Evolving cancer-niche interactions and therapeutic targets during bone metastasis. Nature Reviews Cancer.

[ref-83] Sharma G, Pothuraju R, Kanchan RK, Batra SK, Siddiqui JA (2022). Chemokines network in bone metastasis: vital regulators of seeding and soiling. Seminars in Cancer Biology.

[ref-84] Shiozawa Y, Pedersen EA, Patel LR, Ziegler AM, Havens AM, Jung Y, Wang J, Zalucha S, Loberg RD, Pienta KJ, Taichman RS (2010). GAS6/AXL axis regulates prostate cancer invasion, proliferation, and survival in the bone marrow niche. Neoplasia.

[ref-85] Shuai Y, Huang H (2025). Transcriptional and epigenetic reprogramming, lineage plasticity and therapy resistance in prostate cancer. Journal of the National Cancer Center.

[ref-86] Shupp AB, Kolb AD, Mukhopadhyay D, Bussard KM (2018). Cancer metastases to bone: concepts, mechanisms, and interactions with bone osteoblasts. Cancers.

[ref-87] Singh DK, Patel VG, Oh WK, Aguirre-Ghiso JA (2021). Prostate cancer dormancy and reactivation in bone marrow. Journal of Clinical Medicine.

[ref-88] Singh S, Sarma DK, Verma V, Nagpal R, Kumar M (2023). From cells to environment: exploring the interplay between factors shaping bone health and disease. Medicina.

[ref-89] Sophia Fox AJ, Bedi A, Rodeo SA (2009). The basic science of articular cartilage: structure, composition, and function. Sports Health.

[ref-90] Sottnik JL, Dai J, Zhang H, Campbell B, Keller ET (2015). Tumor-induced pressure in the bone microenvironment causes osteocytes to promote the growth of prostate cancer bone metastases. Cancer Research.

[ref-91] Tang R, Luo S, Liu H, Sun Y, Liu M, Li L, Ren H, Angele MK, Börner N, Yu K, Guo Z, Yin G, Luo H (2025). Circulating tumor microenvironment in metastasis. Cancer Research.

[ref-92] Taveira-Barbosa J, Morais S, Garcia T, Bento MJ, Lunet N (2025). Second primary cancers among males with a first primary prostate cancer: a population-based study in northern Portugal. Clinical and Experimental Medicine.

[ref-93] Tiede-Lewis LM, Dallas SL (2019). Changes in the osteocyte lacunocanalicular network with aging. Bone.

[ref-94] Trivedi T, Pagnotti GM, Guise TA, Mohammad KS (2021). The role of TGF-β in bone metastases. Biomolecules.

[ref-95] Ullah TR (2019). The role of CXCR4 in multiple myeloma: cells’ journey from bone marrow to beyond. Journal of Bone Oncology.

[ref-96] van der Deen M, Akech J, Wang T, FitzGerald TJ, Altieri DC, Languino LR, Lian JB, van Wijnen AJ, Stein JL, Stein GS (2010). The cancer-related Runx2 protein enhances cell growth and responses to androgen and TGFβ in prostate cancer cells. Journal of Cellular Biochemistry.

[ref-97] Verbruggen SW (2024). Role of the osteocyte in bone metastasis—the importance of networking. Journal of Bone Oncology.

[ref-98] Vlaeminck-Guillem V (2018). Extracellular vesicles in prostate cancer carcinogenesis, diagnosis, and management. Frontiers in Oncology.

[ref-99] Wang Y, Chen S, Fan W, Zhang S, Chen X (2025a). Cell ratio-dependent osteoblast-endothelial cell crosstalk promoting osteogenesis-angiogenesis coupling via regulation of microfluidic perfusion and paracrine signaling. Micromachines.

[ref-100] Wang N, Docherty FE, Brown HK, Reeves KJ, Fowles ACM, Ottewell PD, Dear TN, Holen I, Croucher PI, Eaton CL (2014). Prostate cancer cells preferentially home to osteoblast-rich areas in the early stages of bone metastasis: evidence from in vivo models. Journal of Bone and Mineral Research.

[ref-101] Wang Z, Ren L, Li Z, Qiu Q, Wang H, Huang X, Ma D (2025b). Impact of different cell types on the osteogenic differentiation process of mesenchymal stem cells. Stem Cells International.

[ref-102] Wang M, Xia F, Wei Y, Wei X (2020). Molecular mechanisms and clinical management of cancer bone metastasis. Bone Research.

[ref-103] Wang W, Yang X, Dai J, Lu Y, Zhang J, Keller ET (2019). Prostate cancer promotes a vicious cycle of bone metastasis progression through inducing osteocytes to secrete GDF15 that stimulates prostate cancer growth and invasion. Oncogene.

[ref-104] Wani SA, Qudrat S, Zubair H, Iqbal Z, Gulzar B, Aziz S, Inayat A, Safi D, Kamran A (2025). Role of osteoclast inhibitors in prostate cancer bone metastasis; a narrative review. Journal of Oncology Pharmacy Practice.

[ref-105] Wells KV, Krackeler ML, Jathal MK, Parikh M, Ghosh PM, Leach JK, Genetos DC (2023). Prostate cancer and bone: clinical presentation and molecular mechanisms. Endocrine-Related Cancer.

[ref-106] Widyadharma IPE, Tertia C, Vania A, Tiffani P, Wiratnaya IGE (2024). The effect of denosumab vs. zoledronic acid in preventing skeletal-related events, including pain-related bone metastasis: a systematic review. Advances in Psychiatry and Neurology.

[ref-107] Wong SK, Mohamad N-V, Giaze TR, Chin K-Y, Mohamed N, Ima-Nirwana S (2019). Prostate cancer and bone metastases: the underlying mechanisms. International Journal of Molecular Sciences.

[ref-108] Woo S-M, Paek K, Yoon YM, Kim H, Park SI, Kim JA (2025). Development of a BMU-on-a-chip model based on spatiotemporal regulation of cellular interactions in the bone remodeling cycle. Materials Today Bio.

[ref-109] Wu M, Chen G, Li Y-P (2016). TGF-β and BMP signaling in osteoblast, skeletal development, and bone formation, homeostasis and disease. Bone Research.

[ref-110] Wu Z, Li W, Jiang K, Lin Z, Qian C, Wu M, Xia Y, Li N, Zhang H, Xiao H, Bai J, Geng D (2024). Regulation of bone homeostasis: signaling pathways and therapeutic targets. Medcomm.

[ref-111] Xu J, Yu L, Liu F, Wan L, Deng Z (2023). The effect of cytokines on osteoblasts and osteoclasts in bone remodeling in osteoporosis: a review. Frontiers in Immunology.

[ref-112] Yao L, Zhang X (2022). Interaction between prostate cancer stem cells and bone microenvironment regulates prostate cancer bone metastasis and treatment resistance. Journal of Cancer.

[ref-113] Ye X, Huang X, Fu X, Zhang X, Lin R, Zhang W, Zhang J, Lu Y (2023). Myeloid-like tumor hybrid cells in bone marrow promote progression of prostate cancer bone metastasis. Journal of Hematology and Oncology.

[ref-114] Yu G, Corn PG, Mak CSL, Liang X, Zhang M, Troncoso P, Song JH, Lin S-C, Song X, Liu J, Zhang J, Logothetis CJ, Melancon MP, Panaretakis T, Wang G, Lin S-H (2024). Prostate cancer–induced endothelial-cell-to-osteoblast transition drives immunosuppression in the bone–tumor microenvironment through wnt pathway–induced M2 macrophage polarization. Proceedings of the National Academy of Sciences of the United States of America.

[ref-115] Yuan S, Hoggard NK, Kantake N, Hildreth BE, Rosol TJ (2023). Effects of dickkopf-1 (DKK-1) on prostate cancer growth and bone metastasis. Cells.

[ref-116] Yu-Ju Wu C, Chen C-H, Lin C-Y, Feng L-Y, Lin Y-C, Wei K-C, Huang C-Y, Fang J-Y, Chen P-Y (2020). CCL5 of glioma-associated microglia/macrophages regulates glioma migration and invasion via calcium-dependent matrix metalloproteinase 2. Neuro-Oncology.

[ref-117] Yu-Lee L-Y, Yu G, Lee Y-C, Lin S-C, Pan J, Pan T, Yu K-J, Liu B, Creighton CJ, Rodriguez-Canales J, Villalobos PA, Wistuba II, de Nadal E, Posas F, Gallick GE, Lin S-H (2018). Osteoblast-secreted factors mediate dormancy of metastatic prostate cancer in the bone via activation of the TGFβRIII-p38MAPK-pS249/T252RB pathway. Cancer Research.

[ref-118] Yuze M, Hu J, Jun L, Cheng X, Tianwen X, Junqiang Z (2025). Osteocytes function as biomechanical signaling hubs bridging mechanical stress sensing and systemic adaptation. Frontiers in Physiology.

[ref-119] Zareba P, Flavin R, Isikbay M, Rider JR, Gerke TA, Finn S, Pettersson A, Giunchi F, Unger RH, Tinianow AM, Andersson S-O, Andrén O, Fall K, Fiorentino M, Mucci LA, Transdisciplinary Prostate Cancer Partnership (ToPCaP) (2017). Perineural invasion and risk of lethal prostate cancer. Cancer Epidemiology, Biomarkers & Prevention.

[ref-120] Zarrer J, Taipaleenmäki H (2024). The osteoblast in regulation of tumor cell dormancy and bone metastasis. Journal of Bone Oncology.

[ref-121] Zhang Y, Alexander PB, Wang X-F (2017). TGF-β family signaling in the control of cell proliferation and survival. Cold Spring Harbor Perspectives in Biology.

[ref-122] Zhang W, Bado I, Wang H, Lo H-C, Zhang XH-F (2019). Bone metastasis: find your niche and fit in. Trends in Cancer.

[ref-123] Zhao D, Hua R, Riquelme MA, Cheng H, Guda T, Xu H, Gu S, Jiang JX (2022). Osteocytes regulate bone anabolic response to mechanical loading in Male mice via activation of integrin α5. Bone Research.

[ref-124] Zhao X, Liu S, Zou Z, Liang C (2025). Global, regional, and national prevalence of prostate cancer from 1990 to 2021: a trend and health inequality analyses. Frontiers in Public Health.

[ref-125] Zhao E, Wang L, Dai J, Kryczek I, Wei S, Vatan L, Altuwaijri S, Sparwasser T, Wang G, Keller ET, Zou W (2012). Regulatory T cells in the bone marrow microenvironment in patients with prostate cancer. Oncoimmunology.

[ref-126] Zheng D, Decker KF, Zhou T, Chen J, Qi Z, Jacobs K, Weilbaecher KN, Corey E, Long F, Jia L (2013). Role of WNT7B-induced noncanonical pathway in advanced prostate cancer. Molecular Cancer Research.

[ref-127] Zhu S, Chen W, Masson A, Li Y-P (2024). Cell signaling and transcriptional regulation of osteoblast lineage commitment, differentiation, bone formation, and homeostasis. Cell Discovery.

